# Social media use for coping with stress and psychological adjustment: A transactional model of stress and coping perspective

**DOI:** 10.3389/fpsyg.2023.1140312

**Published:** 2023-03-21

**Authors:** Xiaobei Sun, Benjamin J. Li, Heng Zhang, Guanwen Zhang

**Affiliations:** ^1^School of Journalism and Communication, Shandong University, Jinan, China; ^2^Wee Kim Wee School of Communication and Information, Nanyang Technological University, Singapore, Singapore; ^3^School of Journalism and Communication, Shandong Normal University, Jinan, China

**Keywords:** psychological well-being, social media, COVID-19, coping strategies, psychological adjustment, perceived stress

## Abstract

**Introduction:**

The psychological well-being of individuals has become an essential issue during the global pandemic. As a pervasive activity for individuals to pull through COVID-19, social media use may play a role in psychological well-being. Drawing on the transactional model of stress and coping, the current study investigated the relationships between COVID-19-related stressors and the use of social media to facilitate specific coping strategies. We further investigated how social media coping strategies impact users’ psychological adjustment.

**Methods:**

We collected the data from 641 quarantined residents through a two-wave survey that was conducted in two cities in China during government-mandated lockdowns.

**Results:**

The results showed that perceived COVID-19 stress was related to the intensity of social media use for problem-focused coping, socioemotional coping, and mental disengagement. In addition, individuals’ psychological adjustment was positively associated with social media use for socioemotional coping and mental disengagement while negatively related to problem-focused coping. Age was also found to be a moderator of the relationship between socioemotional coping and psychological adjustment.

**Discussion:**

To relieve pandemic-related stress, individuals can actively utilize social media to implement various coping strategies. However, coping activities with social media may not always induce psychological benefits. By revealing the different levels of psychological adjustment among social media users with specific coping strategies, the current research enriched the literature on the effects of social media use on mental health. Findings from this study suggest the need for the prudent use of social media to cope with public health crises.

## 1. Introduction

In the post-COVID world, many countries have lifted COVID-19-related policies, and most have returned to their pre-pandemic lifestyles, such as Israel (Keisari et al., 2022) and Thailand (Suksatan et al., 2022). While this hints toward some resolution of the epidemiologic crisis caused by COVID-19, the mental health crisis of COVID-19 may have a lasting impact on people’s health (Mmanga et al., 2023). Studies have demonstrated that COVID-19 can result in a wide range of psychological issues, such as anxiety (Lee et al., 2020) and depression (Mahmud et al., 2021). Evidence from unfolding research suggests that the psychological well-being of individuals during public health emergencies should be a key priority for policymakers and health practitioners.

With past research showing a significant relationship between perceived stress and functional impairment (Manning et al., 2021), it is likely that the perception of stress during COVID-19 could be a key cause of poor psychological well-being. Our experience of COVID-19 has resulted in researchers and policymakers to study how we can reduce harm to psychological well-being by helping individuals to cope with stress triggered by COVID-19 more effectively. Demand for social media skyrocketed as a result of government-mandated lockdowns, leading some researchers to suggest that one benefit from using social media during the COVID-19 period was its ability to reduce users’ perceived stress levels (Zhao and Zhou, 2021; Xie et al., 2022; Buodo et al., 2023). Nonetheless, conclusions regarding the psychological effects of social media use during COVID-19 remain mixed. Some research showed that social media use could help individuals to avoid adverse mental states and decrease negative emotions, signifying its potential to enhance mental health and psychological well-being during COVID-19 (Karakose et al., 2022; Orsolini et al., 2022). On the other hand, using social media as a way to manage the stressful environments triggered by COVID-19 may result in negative outcomes such as psychological exhaustion and information overload (Liu et al., 2021; Wu and Pei, 2022), ultimately impairing users’ psychological well-being (Li and Khan, 2022). However, these conclusions can be attributed to researchers often taking a simplistic view of social media activities, such as the amount of user self-disclosure or time spent online, without taking into account the multidimensional nature of social media, which can encompass factors such as social interaction, information diffusion, and amusement (Islam et al., 2020a; Apuke and Omar, 2021; Zhen et al., 2021). Coping strategies, defined as the purposes or goals of coping activities, involve various social media activities that individuals under lockdown or social isolation may use (Bae, 2022). Individuals may engage in different social media activities, such as searching for information or seeking for social support, which lead to different coping strategies that can help them manage their stress levels (Wolfers and Schneider, 2021). Hence, it is necessary to investigate how the use of social media during COVID-19 leading to distinct coping strategies may foster different psychological outcomes.

Additionally, this study examined the links between social media use during COVID-19 and psychological adjustment. Psychological adjustment refers to one’s ability to keep distress at a minimum in order for positive function in daily life (Chen and Bonanno, 2020; Cruz et al., 2020). Individuals who report higher psychological adjustment typically exhibit more positive psychological well-being. They often can adapt more effectively to stressful environments and are hence less likely to experience mental disorders and negative emotions (Bantjes and Kagee, 2018; Samios, 2018). Moreover, previous research showed that pandemic-related stress negatively affects psychological adjustment (Ellis et al., 2020). Therefore, investigating the relationship between social media use during COVID-19 and psychological adjustment is crucial for developing health interventions that may reduce the adverse mental health effects resulting from the pandemic.

### 1.1. Transactional model of stress and coping

We employed the transactional model of stress and coping (TMSC) as the guiding theory for this study. TMSC proposes that an individual’s appraisal of environmental stimuli as being stressful will lead to coping strategies, which consequently will compel psychological changes within the individual as an outcome of coping with stress (Lazarus and Folkman, 1984, p. 19). Past studies have used TMSC to understand how people use social media to cope with stress resulting from COVID-19 (Bae, 2022; Xie et al., 2022), hence TMSC appears to be an excellent fit for the research context of this study. In line with TMSC, we predict that individuals will interpret COVID-19 as a stressful issue. This will then lead to various coping strategies manifested through social media use (e.g., use of social media for accomplishing problem-focused coping strategy), which will subsequently result in either high or low psychological adjustment.

According to TMSC, coping strategies are implemented when the surrounding environment is seen as stressful (Folkman and Lazarus, 1980). Prior research identified three coping strategies, problem-focused, socioemotional, and mental disengagement, that individuals employ through Internet use in the face of negative life events (van Ingen et al., 2016; Khoo and Yang, 2021). Problem-focused coping refers to the gathering of helpful information for follow-up actions in dealing with problems (Wolfers and Schneider, 2021). Socioemotional coping involves actively participating in online activities, such as seeking social support, to mitigate negative stress-related emotions (Bae, 2022). Mental disengagement is characterized by using the Internet to mentally distract oneself from the stress one is facing (Kırcaburun and Griffiths, 2019; Khoo and Yang, 2021).

Individuals may use social media for problem-focused coping in response to COVID-19-related pressures. The collective fear of COVID-19 creates stress among individuals (Erbiçer et al., 2021). Research shows that individuals with higher COVID-19 fear were more likely to engage in COVID-19-related information-seeking behavior (Brown et al., 2021). Social media can be a key resource for many individuals as it allows them to access the most recent information regarding the virus at their convenience (Liu, 2020). Individuals who employ problem-focused coping through assessing social media information expected to have lower uncertainty toward the pandemic (Bae, 2022) and display more confidence in planning appropriate actions such as vaccination (Manganello et al., 2022).

Next, social isolation may occur due to lockdowns, with the loss of face-to-face interaction resulting in negative emotions (Qiu et al., 2020). As individuals can choose to be fully or partially anonymous on social media, they may view these platforms as safe places to freely express their negative emotions during the pandemic (Gu et al., 2022). Moreover, for individuals who perceive their offline realities as unfulfilling due to social restrictions, social media can serve as an alternative avenue for them to both maintain existing social bonds and form new ones (O’Day and Heimberg, 2021). Hence, socioemotional coping can be achieved as individuals share their painful experiences with others on social media.

Finally, many individuals, particularly parents, face unemployment or financial strain due to COVID-19. As a result, they often assume additional work obligations during this period, which can cause exhaustion and burnout (Amanullah and Ramesh, 2020; Islam et al., 2020b). These negative outcomes may impede a person from controlling the already stressful situation caused by pandemic (AlJhani et al., 2021). Consequently, perceived stress from COVID-19 may potentially induce individuals to find psychological escape through social media, using mental disengagement as a coping strategy. Social media provides a variety of opportunities to escape from reality temporarily. For example, reading memes on *Facebook* or watching funny *Tiktok* videos may help to distract from the stress one is feeling (Karakose et al., 2022).

TMSC suggests that coping strategies may play a role in psychological adjustment as adaptive coping strategies have been found to increase resilience and decrease the risks of mental problems caused by stress (Bae, 2022; Xie et al., 2022). In addition, research suggests that effective stress coping may contribute to an individual’s ability to adjust to discomfort (Fluharty et al., 2021; Gregus et al., 2022). Hence, coping strategies may be important indicators of psychological adjustment toward stress. Information seeking was linked to poor mental health outcomes during COVID-19, with individuals demonstrating stronger information seeking behavior more likely to be diagnosed with higher anxiety (Wilding et al., 2022). Moreover, emotional support was positively related to environmental mastery regarding COVID-19, resulting in better psychological well-being (Dumciene and Pozeriene, 2022). This suggests that seeking support from others may result in more positive psychological adjustment in the long run. One study revealed that avoidance coping, similar to mental disengagement, is significantly associated with increased psychological symptoms (Türk et al., 2021). Despite past studies suggesting the plausible links between coping strategies and psychological adjustment, it is worth noting that whether a coping strategy is assessed to be adaptive or maladaptive should depend on the individual’s reaction to their distinct coping efforts in a specific situation (Wolfers and Schneider, 2021). In addition, Wolfers and Utz (2022) proposed the situational fit of using social media for stress management, suggesting that even the same coping strategy might relate differently to individuals’ well-being depending on the context of media use. Hence, we intend to examine how social media coping strategies relate to psychological adjustment during the pandemic.

### 1.2. Age as a moderating factor

Past research suggests that age may be important to consider with regard to preferred social media coping strategies. A study by Losada-Baltar et al. (2021) showed that older and younger adults in Spain demonstrated differences in coping strategies during the COVID-19 pandemic. Other studies revealed that adolescents and the elderly vary in their preference of social media coping strategies (Minahan et al., 2021; Khalili-Mahani et al., 2022). Different age groups may also utilize social media to varying degrees. In contrast to the older generation, young people appear to have an exceptionally high degree of dependence on social media. For instance, with respect to how people obtain health-related information from the media, young people are more likely to utilize social media, while older people are more likely to use traditional media such as television (Williams et al., 2022).

Psychological adjustment is a dynamic process, and as one increases in age, they often adapt better to frustrations and hardships in their lives (Heckhausen et al., 2019). Although older people are at a higher risk of infection-related complications, several studies showed that they responded better to stress generated by the outbreak of COVID-19 (Kuhn et al., 2021; Daly et al., 2022). In contrast, adolescents and young adults seem more susceptible to psychological distress during the pandemic (Vacchiano, 2022). Given that people of different ages may vary in both their expectations regarding social media use and ability to adjust to COVID-19, we are keen to explore if age is a potential moderator of the relationships between social media coping strategies and psychological adjustment.

### 1.3. The current study

**Figure 1:**
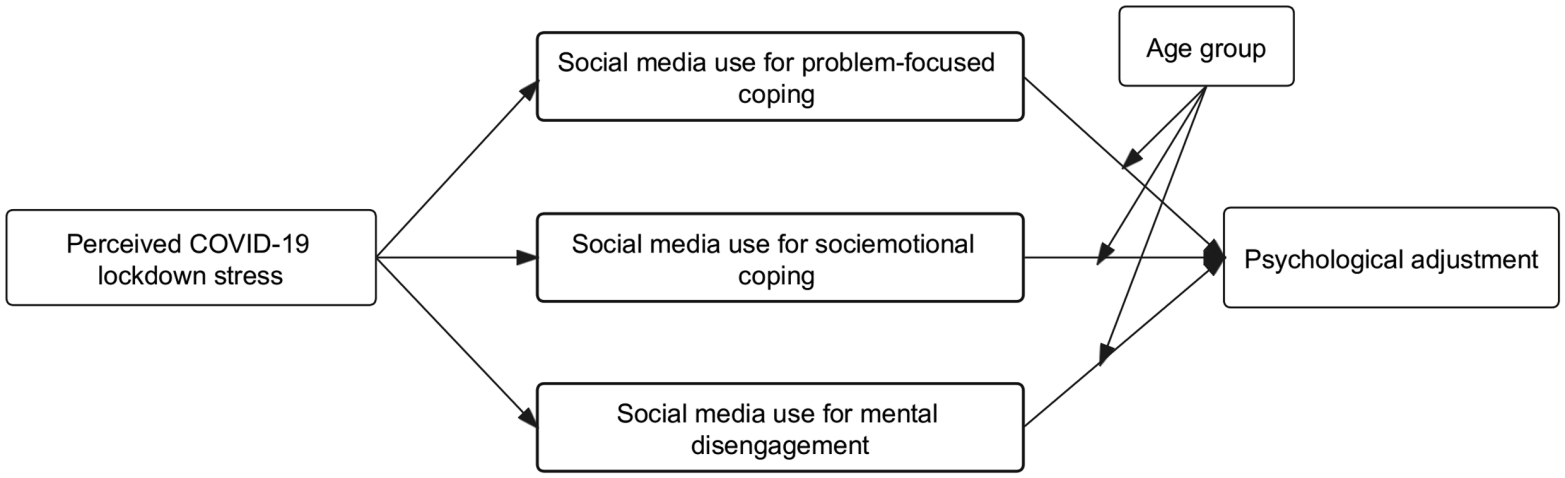
Research model in our study.

Drawing upon TMSC as the overarching theoretical framework, this study aims to investigate how perceived COVID-19 stress relates to social media coping strategies (RQ1), how social media coping strategies affect psychological adjustment (RQ2), and if there are age differences in the relationship between social media coping strategies and psychological adjustment (RQ3). The research model is presented in Figure 1.

## 2. Materials and methods

### 2.1. Data collection

The study was conducted during a government-mandated lockdown. As COVID-19 is a key concern for individuals during this period, this will more accurately reflect their psychological well-being during the pandemic. A two-wave survey was conducted in a midwestern city (city A) and an eastern city (city B) in China. Both cities are provincial capital cities with vast geographic areas and large populations. The implementation of the lockdown in these two cities was crucial for preventing further spread of COVID-19 in China. It lasted more than 1 month in City A, from December 2021 to January 2022. The mandatory quarantine in City B for areas infected or at risk of infection lasted over a month, from April 2022 to May 2022. Hence, these two cities were appropriate for the study.

E-questionnaires were sent to respondents slightly after 2 weeks after the lockdown announcements in both cities when respondents were still under lockdown. To identify target respondents, two screening questions, including “Have you been under lockdown for more than 2 weeks from now?” and “Will you still be under lockdown for a period of time?” were presented to respondents at the beginning of the survey. Respondents who answered “No” to any of the two questions were excluded from the survey.

A description was provided at the beginning of the questionnaire, which explained the study purpose, provided privacy assurances, and listed instructions for filling out the questionnaire. All participants provided informed consent and were given monetary compensation for their time.

Our data collection yielded 690 responses in total. Invalid questionnaires, including those by respondents who consecutively selected multiple identical values across all main variables or those with a high number of missing responses, were excluded during data cleaning. The final sample comprised 641 responses, of which 337 were from city A and 304 from city B.

### 2.2. Measures

#### 2.2.1. Social media coping strategies

To measure social media copings strategies, 14 items were adapted from the inventory of online coping (van Ingen et al., 2016). As the original items were used to measure online coping after negative life events, the wording was modified to fit the context of our study. Specifically, words regarding online use, such as “online” and “the Internet,” were replaced with “social media use.” Additionally, we underlined the word “lockdown” as the study context was important to highlight to our respondents. Respondents were instructed to report their intensity to use social media to manage the lockdown by answering 14 items on a five-point Likert scale ranging from 1 (does not apply to me at all) to 5 (applies to me very much). Six items were relevant to problem-focused coping, such as “I use social media to do something about the lockdown” and “I use social media to improve my situation during the lockdown” (*α* = 0.80). Six items were related to socioemotional coping, such as “I obtain emotional support from others through social media” and “I receive comfort and understanding from someone through social media” (*α* = 0.77). Mental disengagement was measured using two items: “I turn to social media to take my mind off the lockdown” and “I use social media to avoid thinking about the lockdown” (*α* = 0.75).

#### 2.2.2. Psychological adjustment

The current study used the Brief Adjustment Scale-6 (Cruz et al., 2020) to assess respondents’ psychological adjustment (e.g., “To what extent have you felt irritable, angry, and/or resentful this week?”). The Brief Adjustment Scale-6 was previously tested for its validity in the context of COVID-19 (Kablinger et al., 2022). All six items on the scale are scored based on a 5-point scale ranging from 1 (not at all) to 5 (extremely). Items were reverse scored, with higher scores indicating greater psychological adjustment (*α* = 0.94).

#### 2.2.3. Perceived COVID-19 stress

To assess perceived COVID-19 stress, we adopted a measure used in a previous study on SARS, which provided a general classification of pandemic-related stressors (Main et al., 2011). This includes dimensions such as self, family, friend, acquaintance, and information stressors. More recently, this measure was used to assess COVID-19-related stressors (Li et al., 2020; Han et al., 2021; Wang et al., 2022). Six items in the original scale were used as they were relevant to the COVID-19 lockdown, such as “confirmed or suspected infection” and “lack food.” Two additional items, “significant reduction of family income” and “important things in life or work have been postponed or canceled,” were included in our questionnaire based on interviews with individuals affected by the COVID-19 lockdown. Participants indicated either “yes” (coded as 1) or “no” (coded as 0) in response to whether they experienced each of these stressors. Perceived COVID-19 stress indexes were computed based on the total number of events endorsed, and ranged from zero to eight. A higher score indicates a higher level of perceived COVID-19 stress.

#### 2.2.4. Age groups

We classified our respondents into three age groups based on a previous segmentation used in a relevant COVID-19 study:18–30 years old, 31–59 years old, and over 60 (Wu et al., 2020). This classification is relatively in line with current definitions of young, middle, and old age groups. All participants were between the ages of 18 and 70 in this study, with 215 aged between 18 and 30 years (33.5%), 385 aged between 31 and 59 years (60.1%), and 41 aged between 60 and 70 (6.4%).

#### 2.2.5. Covariates

We explored several demographic variables as covariates, including gender, geographical location, education level, and daily social media use duration (see Table 1). The sample comprised 68.5% females, while the regional distribution of the samples was relatively fair, with city A accounting for 52.6% of the sample. Most respondents received senior high school education (61.9%), with undergraduate or above (25.3%) and Junior high school (12.2%) constituting the next major education levels. Respondents primarily use social media for over 4 h a day (44.5%), followed by 1–2 h (18.2%) and 2–4 h (16.4%). The main questionnaire for the study is available as Supplementary material.

**Table 1:** Demographic information of participants (*N* = 641).

	Region					*Total*
	City A		City B			
*Gender*						
Male	111		91			202
Female	226		213			439
*Total* (%)	337 (52.6%)		304 (47.4%)			
	Education					
	Primary school	Junior high school	Senior high school	Undergraduate or above		
*Gender*						
Male	3	17	124	58		202
Female	1	61	273	104		439
*Total* (%)	4 (0.6%)	78 (12.2%)	387 (61.9%)	162 (25.3%)		
	Daily social media use duration					
	<0.5 h	0.5-1 h	1–2 h	2–4 h	> 4 h	
*Gender*						
Male	10	30	37	30	95	202
Female	23	71	80	75	190	439
*Total* (%)	33(5.1%)	101(15.8%)	117(18.2%)	105(16.4%)	285(44.5%)	

### 2.3. Statistical analysis

The reliance on a single survey may result in a high coverage error, hence the results based on these samples cannot be generalized easily (Ha et al., 2015; Marquart, 2017). To improve sample heterogeneity, we conducted the surveys at different times in the two cities. This research design allows us to be more confident in drawing a generalizable conclusion from the results. Therefore, the data collected in the two cities were put together for data analysis rather than separately compared.

We primarily employed descriptive, correlation, and regression analyses using *SPSS* 25.0 (IBM Corp, 2017). In a preliminary analysis, we calculated bivariate correlations among variables of interest, in which a two-tailed *value of p* smaller than 0.05 indicated the presence of statistical significance. A descriptive analysis of perceived COVID-19 stress was conducted as well.

Regression analyses were conducted to explore the relationships between perceived COVID-19 stress and social media coping strategies (RQ1). As gender, region, and daily social media use duration were significant demographic variables in our bivariate correlation analyses, they were included as covariates. We then conducted hierarchical regression analysis to explore the relationship between social media coping strategies and psychological adjustment (RQ2) and whether the age group moderates these associations (RQ3). In the first block, variables representing social media coping strategies were entered to investigate the extent to which they explain psychological adjustment outcomes. To examine moderation effects, interaction terms were generated by multiplying the mean-centered values of the respective main effect variables. We then included age group and the interaction terms between the mean-centered variables of social media coping strategies and age group in sequence.

## 3. Results

### 3.1. Preliminary analysis

As shown in Table 2, most of the demographic variables of participants appear to significantly correlate with the key variables with the exception of education. Social media coping strategies were significantly related to gender, region, and daily social media use duration (all *p* < 0.05). However, psychological adjustment was not significantly associated with the demographic variables.

**Table 2:** Bivariate correlations among covariates and key variables.

	SMUPC	SMUSC	SMUMD	PA
*Covariates*				
Gender	−0.10^**^	−0.06	−0.05	0.02
Region	−0.15^***^	−0.17^***^	−0.20^***^	−0.03
Education	−0.06	−0.07	−0.03	−0.03
Daily social media use duration	−0.11^**^	−0.06	−0.09^*^	0.03

^*^p < 0.05, ^**^p < 0.01, ^***^p < 0.001. SMUPC: Social media usage for problem-focus coping; SMUSC: Social media usage for socioemotional coping; SMUMD: Social media usage for mental disengagement; PA: Psychological adjustment.

### 3.2. Descriptive statistics of perceived COVID-19 stress

**Figure 2:**
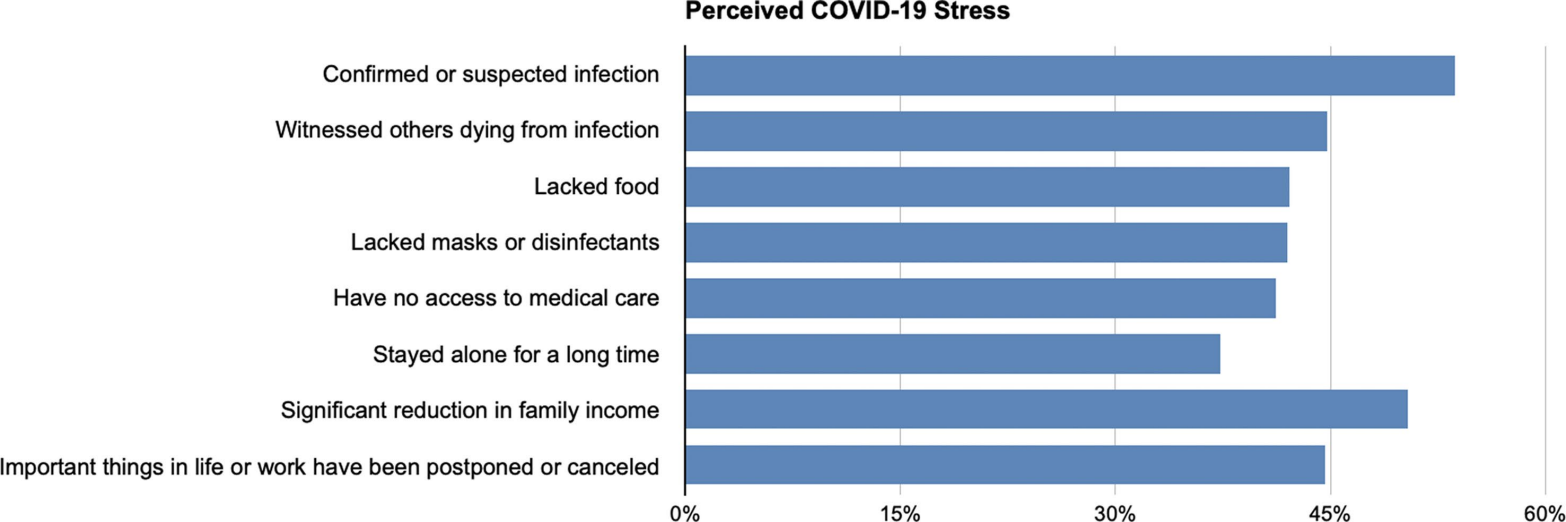
Descriptive statistics for perceived COVID-19 stress.

Figure 2 presents the descriptive statistics of perceived COVID-19 stress. 53.7% of respondents report having suffered from “Confirmed or suspected infection” during the lockdown. 50.4% of respondents felt stressed from the “Significant reduction in family income.” 44.8% of respondents agreed that “Witnessing others dying from infection” was one of the significant stressors during the lockdown. “Important things in life or work have been postponed or canceled” was reported as a stressor for 44.6% of respondents. Over 35% of our respondents reported experiencing all of the stressors.

### 3.3. Relationships between perceived COVID-19 stress, social media coping strategies, and psychological adjustment

Table 3 shows the regression analysis results for the associations between perceived COVID-19 stress and social media coping strategies. Significant positive associations were revealed between perceived COVID-19 stress and social media coping strategies, providing answers for RQ1. Specifically, after controlling for gender, region, and daily social media use duration, perceived COVID-19 stress was positively related to the intensity of social media use for problem-focused coping (*β* = 0.18, *p* < 0.01), socioemotional coping (*β* = 0.22, *p* < 0.001), and mental disengagement (*β* = 0.21, *p* < 0.001).

**Table 3:** Coefficients of regression analysis predicting social media coping strategies.

	DV: SMUPC		DV: SMUSC		DV: SMUMD	
Variable	*β*	Δ*R*^2^	*β*	Δ*R*^2^	*β*	Δ*R*^2^
*Block 1: Control variables*						
Gender	−0.10^*^	0.03^***^	−0.06	0.03^***^	−0.04	0.04^***^
Region	−0.13^**^		−0.16^***^		−0.19^***^	
Daily social media use duration	−0.09^*^		−0.04		−0.06	
*Block 2: Predictor*						
Perceived COVID-19 stress	0.18^**^	0.05^***^	0.22^***^	0.05^***^	0.21^**^	0.05^***^

SMUPC: Social media usage for problem-focus coping; SMUSC: Social media usage for socioemotional coping; SMUMD: Social media usage for mental disengagement. *N* = 641. ^*^p < 0.05, ^**^p < 0.01, ^***^p < 0.001.

Analyses showed differences in the correlations of social media coping strategies to psychological adjustment, which answered RQ2. In particular, using social media for problem-focused coping was negatively associated with psychological adjustment (*β* = −0.16, *p* < 0.01). On the other hand, social media usage for socioemotional coping (*β* = 0.21, *p* < 0.001) and mental disengagement (*β* = 0.23, *p* < 0.001) were significantly positively related to psychological adjustment (see Table 4).

**Table 4:** Coefficients of regression analysis predicting psychological adjustment.

	Psychological adjustment		
	*β*	*β*	*β*
SMUPC	−0.16^**^	−0.15^**^	−0.15^**^
SMUSC	0.21^***^	0.21^***^	0.21^***^
SMUMD	0.23^***^	0.23^***^	0.24^***^
Incremental *R*^2^ (%)	8.70^***^		
*Age group as the moderator*			
Age group		−0.03	−0.29
Incremental *R*^2^ (%)		0.00	
SMUPC × Age group			−0.03
SMUSC × Age group			−0.09*
SMUMD × Age group			−0.00
Incremental *R*^2^ (%)			1.3*

SMUPC: Social media usage for problem-focus coping; SMUSC: Social media usage for socioemotional coping; SMUMD: Social media usage for mental disengagement. *N* = 641. ^*^p < 0.05, ^**^p < 0.01, ^***^p < 0.001.

### 3.4. Age group as a moderator

We further investigated age group as a moderator in the research model to answer RQ3. As shown in Table 4, age group was a significant moderator only for the use of social media for socioemotional coping (*β* = −0.09, *p* < 0.05). Specifically, age group negatively moderated the relationship between social media use for socioemotional coping and psychological adjustment. This suggests that the positive association between socioemotional coping and psychological adjustment appears weaker among older individuals than young- and middle-aged individuals.

## 4. Discussion

### 4.1. Key findings

Several significant findings were uncovered in this study. First, perceived COVID-19 stress can drive people to use social media for problem-focused coping, socioemotional coping, and mental disengagement. This implies that during COVID-19 lockdowns, people used social media extensively to cope with stress. The more pressure people faced because of a lockdown, the more likely they were to use social media to relieve stress. Such findings are consistent with past research which suggest higher use of social media among those who were under lockdowns than those who were not (Yue et al., 2021). As previously discussed, social media use for problem-coping may be demonstrated through individuals searching for pertinent information on social media. Even though individuals may mistrust the accuracy of COVID-19-related information shared on social media (Neely et al., 2021), our findings show that social media remains an outlet that were utilized to manage stress.

In line with previous research, a positive relationship was found between perceived COVID-19 stress and social media use for socioemotional coping. Social media use plays a connecting role to the outside world for individuals under lockdown. Especially during times of social isolation, using social media to maintain social relations is an effective way for individuals to accumulate social capital (Boursier et al., 2020), itself found to be a key prerequisite for social support (Wolfers and Utz, 2022). Hence, it is unsurprising to observe a prevalence of seeking social support from online and offline social networks through social media among individuals under lockdown (Saud et al., 2020).

Additionally, perceived COVID-19 stress positively correlated with the use of social media for mental disengagement. This is in line with earlier findings which reported individuals under lockdown, in an attempt to mentally disengage from the present situation, tend to seek out nostalgic media content to immerse themselves in past happier memories (Wulf et al., 2022). Moreover, this study also showed that higher perceived levels of COVID-19 stress showed stronger relationships with social media use for mental disengagement than problem-focused coping. This suggests that such disengagement-orientated social media use may be a more common practice during a global health crisis where individuals recognize it is a prolonged battle and are in for the long haul.

We found a significant negative association between social media use for problem-focused coping and psychological adjustment. This may be explained by the unpleasant psychological response to exposure to knowledge about COVID-19. Research showed that COVID-related information consumption and sharing during lockdowns negatively affect individuals’ psychological well-being (Yue et al., 2021). Particularly, misinformation and rumors regarding COVID-19 are widely spread on social media, with clickbait titles and headlines drawing people’s attention when they search for information online (van der Linden et al., 2020). Such misinformation exacerbates their uncertainty over COVID-19 and can result in problematic outcomes such as vaccine aversion (Hou et al., 2021). In contrast, children whose parents corrected misinformation to ensure family members had access to accurate information about COVID-19 reported fewer psychological problems (Gregus et al., 2022). In this sense, misinformation on social media may disrupt the regular functioning of individuals, such that the use of social media for problem-solving prevents individuals from reaching effective psychological adjustment during COVID-19. Besides, cyberchondria may further explain the negative relationship between social media use for problem-focused coping and psychological adjustment. Another potential risk of using social media for problem-coping is cyberchondria, which may be triggered when excessively searching for information about COVID-19 online (Starcevic et al., 2021). Past studies suggested an increasing tendency of cyberchondria among individuals exposed to COVID-19-related information through social media (Laato et al., 2020; Ahorsu et al., 2022). Cyberchondria can increase individuals’ anxiety about COVID-19 (Jungmann and Witthöft, 2020) and impair their psychological adjustment toward the pandemic (Arslan et al., 2022). Considering the potentially negative role of social media, individuals should be cautious when gathering information using social media to manage their response to public health crises.

As compared to problem-focused coping, social media use for socioemotional coping is a crucial component for improving psychological adjustment. This may be explained through the perceived social support resulting from the use of social media for socioemotional coping. Studies conducted during the pandemic showed that adolescents’ psychological well-being increased as perceived social support levels increased (Okado et al., 2021; Kurudirek et al., 2022). Indeed, social media can allow users to feel social support from their family and friends through common activities such as posting and sharing of content, and providing reactions to updates through comments and emojis (Saud et al., 2020). Besides, such a finding reinforces the buffering effect of social support, which may weaken psychological impairment due to stress (Li et al., 2020; Zhen et al., 2021). The perception of social support when using social media for socioemotional coping may enhance individuals’ confidence in dealing with adversity during the fight against COVID-19, thereby increasing psychological adjustment (Hou et al., 2020).

Furthermore, the psychological benefits of using social media for mental disengagement, as revealed in our findings, challenge the generally negative attitudes toward escapism-oriented media use, with scholars suggesting they may lead to problematic behaviors such as internet addiction (Zhao et al., 2023). Our study suggests that functional escapism may have been present during pandemic social media use. Humor coping, such as through the consumption of amusing media content, may satisfy one’s instinctual requirements, hence enhancing their well-being (Eden et al., 2020). Moreover, engaging in hedonic daily activities during stressful periods may protect individuals against steep increases in negative emotions (Romm et al., 2021). Consequently, social media use as a form of mental disengagement may aid in the recovery from stress during social distancing measures, which may supplement other health promoting pastimes, such as going to the movies.

Lastly, younger individuals appear to benefit more from using social media for socioemotional coping regarding their psychological adjustment during lockdown, as compared to their older counterparts. This may be due to significant anxieties among young people as they experience a decline of their social capital as a result of fewer face-to-face encounters. Studies suggest that individuals who were born between 1995 and 2007, otherwise known as Generation Z (Lissitsa and Laor, 2021), report a greater risk of disruption to their social interactions than previous generations, leading to increased psychological discomfort (Vacchiano, 2022). Hence, using social media for expressing emotions toward the pandemic within their social networks and finding validation through these networks may assist young people regain and mobilize their social capital, ultimately helping to resist against related psychological impairment.

### 4.2. Implications

This study extends current research on the effects of social media use on mental health from a stress-coping perspective. Using the TMSC as a framework, we investigated the relationships between individual appraisals of the COVID-19 lockdown and coping strategies relating to social media use and psychological adjustment. We highlighted the mental health implications of relying on social media to cope with stressful situations. We also explored age as a moderator in the relationship between coping strategies and psychological adjustment, shedding light on demographical differences in relation to the underlying mechanism tested in this study.

In practice, our findings on how individuals use social media to maintain psychological well-being suggest certain implications for social media users and platforms. Social media users should realize that they can manage their negative emotions resulting from public health emergencies through devoting attention to social media and seeking support on these platforms. However, they should be open to sources of information other than just social media in order to obtain accurate information. Social media providers should consider increasing resources for responsible gatekeeping regarding health-related information and ramp up efforts to filter misinformation on their platforms.

### 4.3. Limitations and future research

While our study provided valuable insights into the effects of social media use on mental health, there are certain limitations. First, due to the cross-sectional nature of this study, we were restricted to making inferences based on correlations between the variables of interest. Future research can consider adopting a longitudinal approach to investigate the causal relationships between social media use and mental health outcomes during health crises. Second, there is a need for future studies to analyze how individuals use social media after lockdowns. Researchers will be better placed to assess the difference in social media strategies when stress levels are lower in the aftermath of a health crisis. The mental health effects of social media use can be better understood by comparing the psychological adjustments within individuals at different times. Finally, as each country has their own COVID-19 policies, our findings may not be generalizable to other contexts due to the geographical site of our study. Comparing China with other countries affected by the pandemic would provide more comprehensive information regarding the effect of social media use on psychological well-being during health emergencies.

## 5. Conclusion

Our lives have become deeply intertwined with social media during the pandemic. It is necessary to develop a keen awareness of the psychological ramifications of social media use. From our findings, social media appears to facilitate various strategies for coping with pandemic-related stress. Using social media for certain coping strategies may, however, increase the risk of psychological harm among users. There is still much potential for research on how people use social media and its effects on mental health.

## Data availability statement

The raw data supporting the conclusions of this article will be made available by the authors, without undue reservation.

## Ethics statement

Ethical review and approval was not required for the study on human participants in accordance with the local legislation and institutional requirements. The patients/participants provided their written informed consent to participate in this study.

## Author contributions

XS contributed to the overall conception of this study, data collection and analysis, writing—original draft, and writing—revision and editing. BL contributed to validation and writing—revision and editing. HZ contributed to writing—original draft and writing—revision. GZ contributed to study design and validation. All authors contributed to the article and approved the submitted version.

## Conflict of interest

The authors declare that the research was conducted in the absence of any commercial or financial relationships that could be construed as a potential conflict of interest.

## Publisher’s note

All claims expressed in this article are solely those of the authors and do not necessarily represent those of their affiliated organizations, or those of the publisher, the editors and the reviewers. Any product that may be evaluated in this article, or claim that may be made by its manufacturer, is not guaranteed or endorsed by the publisher.
