# Supplementary material for: Social media use for coping with stress and psychological adjustment: A transactional model of stress and coping perspective
Source: Front Psychol. 2023 Mar 21;14:1140312. doi: 10.3389/fpsyg.2023.1140312 (PMC10075314; doi:10.3389/fpsyg.2023.1140312)
Supplement: Supplementary file 1 [file Data_Sheet_1.docx]

Supplementary Material

# Social Media Use during COVID-19 Lockdown Survey

Q1: Regarding your social media use during the lockdown, to what extent does the following statement apply to your actual experience?

| I used social media to do something about the lockdown | 1 = this doesn’t apply to me at all  5 = this applies to me a lot |
| --- | --- |
| I used social media to take action to make the situation of lockdown better |  |
| I came up with a strategy about what to do through consulting social media |  |
| With the aid of social media, I thought hard about what steps to take |  |
| With the aid of social media, I tried to see things in a different light, to make it seem more positive |  |
| With the aid of social media, I looked for something good in what happened |  |
| I got help and advice from other people through social media | 1 = this doesn’t apply to me at all  5 = this applies to me a lot |
| I asked people who had similar experiences on social media what they did |  |
| I got emotional support from others through social media |  |
| I received comfort and understanding from someone through social media |  |
| I said things on social media to let my unpleasant feelings escape |  |
| I expressed my negative feelings on social media |  |
| I turned to social media to take my mind off things | 1 = this doesn’t apply to me at all  5 = this applies to me a lot |
| I tried to do something on social media to think about Covid-19 less |  |

Q2: Please answer the following questions according to your actual experience.

| To what extent you have felt irritable, angry, and/or resentful this week? | 1= not at all 5=extremely |
| --- | --- |
| To what extent you have felt tense, anxious, and/or afraid this week? |  |
| To what extent have you felt unhappy, discouraged, and/or depressed this week? |  |
| How much has emotional distress interfered with feeling good about yourself this week? |  |
| How much has emotional distress interfered with your relationships this week? |  |
| How much has emotional distress interfered with your ability to perform at work, school, etc. |  |

Q3: Do you suffer from the following issues resulting from COVID-19 during the lockdown?

| Confirmed or suspected infection | 0=no  1=yes |
| --- | --- |
| Witnessed others dying from infection |  |
| Lacked food |  |
| Lacked masks or disinfectants |  |
| Had no access to medical care |  |
| Stayed alone for a long time |  |
| Significant reduction in family income |  |
| Important things in life or work have been postponed or canceled |  |

Q4: How long do you use social media daily?

A. < 0.5 h B. 0.5-1h C. 1-2h D. 2-4h E. > 4h

Q5: Your gender is________?

A. Male B. Female

Q6: Your age is_______?

Q7: Your education level is________?

A. Primary school B. Junior high school C. Senior high school D. Undergraduate or above
